# Adhesion in Physiological, Benign and Malignant Proliferative States of the Endometrium: Microenvironment and the Clinical Big Picture

**DOI:** 10.3390/cells7050043

**Published:** 2018-05-16

**Authors:** Emily J. Rutherford, Arnold D. K. Hill, Ann M. Hopkins

**Affiliations:** Department of Surgery, Royal College of Surgeons in Ireland, RCSI Smurfit Building, Beaumont Hospital, Dublin 9, Ireland; erutherford@rcsi.com (E.J.R.); adkhill@rcsi.com (A.D.K.H.)

**Keywords:** adhesion, ICAM-1, VCAM-1, integrins, endometrium, endometriosis, endometrial cancer, microenvironment, inflammation, seed and soil, biomarkers

## Abstract

Although the developments in cellular and molecular biology over the last few decades have significantly advanced our understanding of the processes and players that regulate invasive disease, many areas of uncertainty remain. This review will discuss the contribution of dysregulated cell–cell and cell–matrix adhesion to the invasion in both benign and malignant contexts. Using the endometrium as an illustrative tissue that undergoes clinically significant invasion in both contexts, the adhesion considerations in the cells (“seed”) and their microenvironment (“soil”) will be discussed. We hope to orientate this discussion towards translational relevance for the diagnosis and treatment of endometrial conditions, which are currently associated with significant morbidity and mortality.

## 1. Introduction

Over the last century, as medical science has extended human life expectancy and reduced the burden of infectious diseases, research has turned its attention to the burden of non-infectious conditions, including cancer. Our understanding of the pathophysiology of malignant diseases has accelerated significantly in recent times. However, as populations age and our knowledge about disease manifestations, natural histories and their intricacies expands, it is increasingly clear that there is much yet to be elucidated. The contribution of adhesion biology to malignant disease represents a field of broad interest, with considerable progress made in recent years. However, there are many nuances still to be explored, especially in the context of the cellular microenvironment.

The disease microenvironment frequently drives anatomically-specific expertise in oncology, with breast cancer managed as a different disease entity compared to gastric cancer, despite the likelihood of some pathophysiological overlap. Such overlap is perhaps best viewed through the lens of the progress made to date in understanding the molecular mechanisms and genetics of cancer. However, in applying biological reductionism to the level of cells, molecules and genes, it is important to remember the need to rebuild the complexity of a tumour and its microenvironment in order to contextualise understanding. Accordingly, there has recently been a renewal of popular interest surrounding the interaction between a disease and its site, which is also known as the “seed and soil” hypothesis [1]. This review will discuss the specific molecules involved in physiological adhesion, benign proliferative conditions and malignant invasive disease in the context of microenvironment interactions and the seed and soil hypothesis. In physiological terms, the functioning of adhesion molecules is of paramount importance throughout the body. Their roles include the maintenance of cell architecture [2,3] and the regulation of diverse homeostatic processes, such as haemostasis, angiogenesis, migration and barrier function [4,5]. This review will instead focus on the *pathophysiological* framework within which adhesion molecules participate, concentrating on the endometrium as a model. The microenvironment or “soil” in which these molecules function is of paramount importance in this analysis. In particular, the role of adhesion molecules in the invasive/migratory contexts (benign and malignant) and their interaction with the immune/inflammatory milieu will be addressed. The specific adhesion molecules that will be focused upon are members of the integrins and immunoglobulin superfamilies in light of their fundamental contributions to both cell–matrix and cell–cell adhesion.

## 2. Seed and Soil Hypothesis and the Role of Adhesion Molecules

The seed and soil hypothesis proposes the idea that a match between the disease and its microenvironment is the most important determinant of disease “success”. The microenvironment of any tumour involves a complex interplay between the influence of multiple cell types, both immune and non-immune, in addition to soluble factors, including hormonal influences. This is not a new idea as Paget first raised the concept in the context of breast cancer over a hundred years ago [6]. Both the early radical surgeries [7] and the focus on genetics in the twenty first century [8] represented huge advances in cancer management, but neglected the aforementioned crucial interplay. This interplay was also overlooked in the original concept of how metastases arose, which simply stated that cancer cells entered a one-way traffic flow to anatomically downstream organs (for example in axillary nodal spread of breast cancer). The anatomic model neglected to recognize that metastases typically show a clear preference for certain organs (classically lung, liver, bone and brain), while other nearby organs (such as spleen, kidneys and gut) are frequently spared [9]. Notably, this preference cannot be explained solely by anatomical proximity or vascular supply. Preferential metastasis to certain organs has been demonstrated in an animal model of melanoma [10], whereby the neoplastic lesions grew in pulmonary grafts but not renal grafts. Accordingly, both research and therapeutics must consider the homeostatic environment and its regulation of angiogenesis, tumour growth and survival and cellular invasion [11]. The specific roles of the intracellular and soluble cellular adhesion molecules, integrins and their interaction with the immune system in the context of both tumorigenic and non-tumorigenic invasion of endometrial tissue will be the main focus of this discussion.

As mentioned above, malignant disease is of continuous interest in research and adhesion biology is of particular relevance when one considers its potential contributions to the dysregulation of proliferation and cellular invasion. However, outside of the malignancy–metastasis paradigm, some other pathologies also show a propensity for cellular invasion outside of the “normal” milieu, with endometriosis being one such condition. Endometriosis can be defined as the presence of endometrium-like tissue in the sites outside the uterine cavity, such as the pelvic peritoneum and ovaries [12]. The original hypothesis of “retrograde menstruation” (a reflux of endometrium into the peritoneal cavity during menstruation, where it implants on bowel, bladder, ovaries etc. [13]) has long been accepted as at least one facet of a possible pathogenesis. However, in recent years, it is increasingly recognized that many women undergo retrograde menstruation without ever developing endometriosis [14]. Just as the malignant tumours do not simply spread to the closest possible organ, it has also become apparent that there are many other factors besides the intuitive anatomical spread of the ectopic tissue. The role of inflammation and potential immune system dysregulation is also clinically evident, with associations between endometriosis and, for example, inflammatory bowel disease noted in large-scale studies [15]. Ectopic endometrium has obvious adverse clinical effects, including pain, dyspareunia, and subfertility [14], while the treatment of endometriosis is still suboptimal [16]. With the above-mentioned considerations, it is clear that although endometriosis may not carry the same dramatic consequences as metastatic cancer in terms of mortality, morbidity is still appreciable and as such, this disease deserves thorough scientific consideration. The role of cell adhesion molecules in endometriosis has long been recognised and together with their involvement in malignancy, they will be reviewed below.

## 3. Integrins- Role in Endometriotic Lesions and Subfertility in Endometriosis Patients

Integrins are transmembrane heterodimers composed of α and β subunits [17], which have crucial functions in normal physiology as the main adhesion receptors for the extracellular matrix (ECM). However, they can also participate in cell–cell adhesion [18]. Integrin adhesion receptors play a critical role in “inside-out signalling”, with receptor activation initiated by intracellular signals [19] although signalling can be bidirectional [20,21]. Accordingly, the integrins are adept at undergoing conformational changes to increase affinity for a ligand, clustering to increase avidity or both [20].

Integrins respond to the dynamic factors within the ECM in the modulation of normal physiological proliferation, growth and differentiation [22]. Endometrial integrins play a key part in normal pregnancy as a binding site for the trophoblast attachment glycoproteins [23]. Integrins also have a recognised role in the endometrial changes during a normal menstrual cycle, with dynamic spatial and temporal changes throughout the cycle. In terms of spatial distribution, the integrin subunits, such as α2 and α3, that promote cell–cell adhesion are described to have a pericellular distribution (as opposed to expression on the basement membrane), in accordance with their known role in binding the ECM components collagen and laminin. From a temporal point of view, the expression of the α1 integrin subunit on the glandular epithelium is observed during the secretory phase of the menstrual cycle [24], where in concert with α4, αV and β3 subunits, it is hypothesized to signal the implantation window [25] by which the uterine lining becomes receptive to a blastocyst [26].

Many factors, including pro-inflammatory and pro-fibrotic cytokines, may alter the expression of endometrial integrins in endometriotic disease [27]. TGF-β1 has been shown to increase the adhesion of endometrial stromal cells in vitro in parallel with increased expression of α5, αV, β3 and β5 integrins in ectopic endometrium in endometriosis patients compared to entopic endometrium. This is consistent with other work suggesting increased integrin expression via the TGFβ-1/Smad2 signalling pathway [28]. Increased entopic endometrial mRNA levels of αV integrin and αVβ3 integrins have also been shown in women with endometriosis as compared to healthy controls, which occurs in conjunction with increased peritoneal IL-1β mRNA levels [29]. As the αVβ3 integrin is known to bind to fibronectin [30], it has been proposed that increased levels of this adhesion molecule in endometriotic lesions may facilitate the binding of endometrium to the peritoneum [31]. Comparably, the adhesion molecules have recently been implicated in post-inflammatory tubal ectopic endometrium tissue after chlamydial infection [32]. The increased expression of integrin molecules in the context of inflammation may be one mechanism by which the refluxed endometrial cells implant on the peritoneum and persist in women with endometriosis. In vitro studies have shown that treatment with cytokines increases the integrin-mediated adhesion of endometriotic cells (with TNF-α increasing adhesion to collagen and IL-1 increasing adhesion to laminin and fibronectin) [33]. Such dysregulation of the endometrial integrin expression could potentially contribute to both loss and gain of function. For example, the hypothesis that dysregulated integrin expression reduces fertility has been proposed for a long time, which likely occurs through a combination of mechanisms leading to decreased receptivity of the endometrial lining to a blastocyst [26]. Sex steroids have been shown to increase β3 integrin expression via the homeobox gene HOXA10 [34]. Accordingly, the alterations in sex steroid levels or their metabolism [35], with subsequent effects on integrin expression, may form one mechanism by which the endometriosis patients display subfertility. More recently, it has been shown that αVβ3 integrin expression was significantly lower in the endometrium of women with mild endometriosis [36] and also in those with unexplained infertility [37]. This reinforces the hypothesis that deficiencies in integrin expression may reduce uterine receptivity. To further illustrate this point, the removal of an inflamed Fallopian tube has been shown to increase αVβ3 integrin expression in luminal endometrial epithelium [38] and to improve outcomes for in vitro fertilisation patients [39].

Other authors have compared the integrin expression in ectopic versus entopic endometrium in the patients with confirmed endometriosis [40], with the interesting finding that α3 integrin was absent in the entopic endometrium of all (*n* = 30) patients with endometriosis, but expressed in the corresponding ectopic endometriotic lesions in 15 cases. The dysregulated expression of integrins by endometriotic tissues was hypothesized to explain the refractory nature of the disease as it was envisioned that the continued integrin expression would allow re-establishment of cell–cell and cell–matrix interactions within the peritoneum. One possible contributor to this process of abnormal endometrial cell adherence to the peritoneum is the pro-inflammatory chemokine macrophage migration inhibition factor (MIF) [41]. Specifically, MIF has been shown to upregulate VEGF and αVβ3 integrins in a human endometrial cell line [42]. Clinically, the persistent expression of integrins could partially account for the high recurrence rate seen in endometriosis [43].

Interestingly, although the current treatment options for endometriosis focus on hormonal aetiologies, the aberrant expression of integrins [40] has been shown to occur independent of ovarian steroid levels [44]. Thus, alternative pathways and mechanisms of disease may be of considerable importance in the development of next-generation treatments. It is tempting to speculate that the adhesion molecules may provide attractive therapeutic targets in this regard, given the undesirable menopausal side effects of current standard treatments, such as gonadotropin releasing hormone (GnRH) agonists.

**Figure 1:**
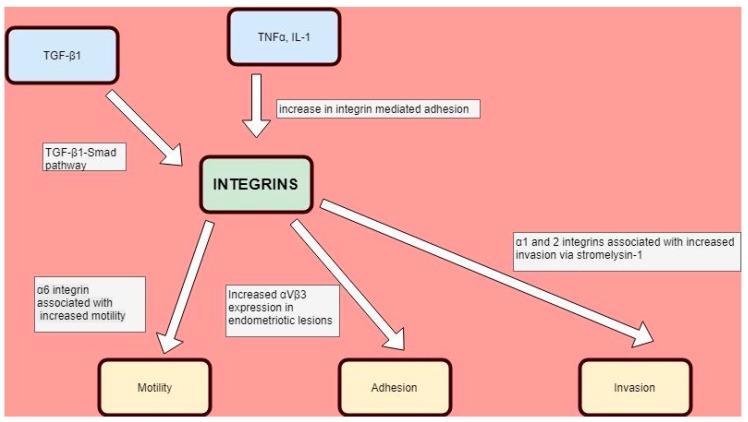
Integrin expression in benign and malignant contexts.

Although endometriosis is a “benign” proliferative disease, the parallel changes in the expression profile of various integrins have also been a recent focus of research attention in malignant disease, specifically with regards to tumour growth, neovascularization and metastatic dissemination [45] (Figure 1). For example, α1 and α2 integrins have been shown to promote invasive behaviour in mouse mammary carcinoma cells via the increased transcription of the matrix metalloproteinase stromelysin 1, while α6 integrins have been linked with increased cell motility [46]. This may have implications for therapeutics in cancers, such as glioblastoma, which are traditionally difficult to treat with conventional means [47].

Although there are many varied ligands for the integrin adhesion receptors, this discussion will next focus on the specific members of the immunoglobulin superfamily (IgSF) of proteins in the context of their function as integrin ligands.

## 4. ICAM-1, sICAM-1 and VCAM-1 in the Immune Context

Integrins can bind to several counter-receptors on many cell types, including members of the IgSF of proteins. Normal endometrial cells express many such members, including the intercellular adhesion molecule-1 (ICAM-1/CD54) and vascular cell adhesion molecule-1 (VCAM-1/CD106). ICAM-1 is a 90-kDa glycoprotein, which consists of five extracellular domains, a single-pass transmembrane domain and a cytoplasmic C terminus [48,49]. ICAM-1 has been recognised to bind to leukocyte-specific integrins [50], while VCAM-1 binds to α4β1, αVβ3 and α4β7 integrins expressed on multiple cell types [51,52,53].

ICAM-1 itself is expressed on multiple immune, epithelial and endothelial cells and binds integrins in a normal physiological state. ICAM-1 acts as a key ligand for leukocyte function antigen-1 (LFA-1), which is an immune system integrin with αLβ2 subunits [54] that promotes leukocyte recruitment in diverse pathological states. The extensive distribution of ICAM-1 in the endometrium and its upregulation during both menstruation [55] and embryo implantation [56] suggests important physiological roles for the molecule in the endometrial setting. Interestingly, preimplantation embryos have been shown to produce the pro-inflammatory cytokine IFN-γ [57], which itself is a stimulator of ICAM-1 expression in the endometrium [58] and elsewhere. Thus, it is plausible that ICAM-1 plays a role in successful endometrial implantation.

The interaction between the adhesion molecules and leukocytes has long been recognised. For example, the neutrophil–endothelium adhesion is mediated by the interaction of β2 integrins with ICAM-1 [59]. Indeed, the role of the ICAM-1–LFA axis in inflammation is of great interest in therapeutics and blocking this interaction has been used as an approach to reduce the activation of T-cells in inflammatory autoimmune diseases and transplant rejection [60]. Increased neutrophil levels and concentrations of human neutrophilic peptides have been noted in the peritoneal fluid of endometriosis patients [61], with a concomitant increase in IL-8 and T-cells. Being consistent with the aforementioned importance of the disease microenvironment, it has been proposed that the cellular interactions with the chronically inflamed endometriotic peritoneal fluid may explain some of the differences between the entopic endometrium and ectopic endometriotic lesions [62]. Diverse factors, including TGF-β, TNF-α, IL-1β and IL-6 that have been shown to upregulate ICAM-1 expression in other contexts, are known to increase endometrial cell adhesion to the peritoneum in an in vitro model of endometriosis [63]. In conjunction with its known role in immune interactions, ICAM-1 has also been posited as a therapeutic target in malignancy and thus, could potentially represent a marker of disease progression and treatment response. ICAM-1 expression on fibroblasts has long been known to be correlated with their exposure to inflammatory mediators, thus increasing intercellular adhesion [50]. In malignant diseases, the production of IL-6 by cancer-associated fibroblasts contributes to tumour–stroma interactions that are involved in many processes, including angiogenesis [64]. Similarly, TNF-α-stimulated upregulation of ICAM-1 in peritoneal mesothelial cells has been described in an animal tumour mode. In this model, treatment with heparin reduced ICAM-1 expression and tumour invasion/adhesion both in vitro and in vivo [65]. Similarly, other authors [66] have shown that ICAM-1 expression in mesothelial cells was increased by pre-incubation with TNF-α or IL-6, with a concurrent increase in tumour adhesion, which was then partially attenuated by the use of a monoclonal antibody against ICAM-1.

Different forms of ICAM-1 have been recognised, with its extracellular domains known to be shed from the cell surface and circulate as soluble ICAM-1 (sICAM-1). Just as endometrial cells constitutively express ICAM-1, sICAM-1 is produced in the normal physiological setting of the endometrium [67]. However, higher levels of sICAM-1 have been noted in patients with a wide range of diseases, including malignancy, autoimmune disease, transplant rejection, asthma and atherosclerosis [48,68,69,70]. Circulating sICAM-1 is easily measured in the blood in vivo and potentially offers a greater level of disease specificity than the inflammatory cytokines that induce its expression. Accordingly, there has been considerable interest in the utility of sICAM-1 as a disease biomarker [71]. However, in the endometrial context, sICAM-1 alone is unlikely to be sufficiently specific for use as a biomarker of endometriosis. However, it may show promise as part of a biomarker panel in conjunction with other markers, including CA-125 [72] and/or soluble VCAM-1 [73].

There is some evidence of hormonal regulation of the shedding of sICAM-1 in the endometrial context, with higher concentrations of sICAM-1 noted in the follicular phase under the influence of estradiol [67,74]. Clinically, it has been reported that the endometrial release of sICAM-1 correlates with the number and score of endometriotic implants [75]. The same group has also suggested that sICAM-1 shedding may directly interfere with the attachment of natural killer or cytotoxic T-cells to endometrial tissue by acting as a decoy site for the binding of LFA-1. This lends further weight to the importance of dysregulated immune-based adhesion events in the pathophysiology of invasion. Others have also noted significantly elevated serum levels of sICAM-1 in endometriosis patients, particularly those with advanced disease [76].

However, the published association between sICAM and endometriosis has not been universally consistent, raising the likelihood that its presence is influenced by the location and stage of disease [77] or by other clinical confounders [78]. Some in vivo studies have noted that the levels of sICAM-1 only *trend* towards a significant increase in women with endometriosis, since the likelihood of other peritoneal sources of sICAM-1 (e.g., the ovaries) complicates the picture [79]. Similarly, there also exists a separate pathway in which IL-1α upregulates the release of sICAM from tumour-associated endothelial cells even in tumours expressing low levels of membrane-bound ICAM-1 [80]. Taken together, these observations suggest a feedback loop in which the cytokine release increases sICAM-1 shedding, which subsequently decreases natural killer (NK) activity via ICAM-1/LFA axis inhibition. This ultimately allows endometriotic lesions to “escape” immunosurveillance. However, this comes with the caveat that multiple sources of sICAM-1 may cloud the clinical picture and complicate its clinical diagnostic utility.

Like ICAM-1, vascular cell adhesion molecule-1 (VCAM-1) is a transmembrane glycoprotein with a key role in endothelial responses to inflammation [81]. Integrin α4β1 (also known as VLA-4) acts as a ligand in VCAM-1-immune cell adhesion [82]. The endothelial expression of VCAM-1 has been shown to be crucial in leukocyte adhesion [83], migration [84] and the triggering of an inflammatory cascade [85]. In the endometrium, VCAM-1 expression can be induced by TNF-α, IL-1α and IFN-γ [86], with resultant increases in leukocyte–endothelial binding. The expression of VCAM-1 on peritoneal mesothelial cells was also found to be associated with endometriosis [87], which creates the “soil” for an endometriotic seed.

Similar to ICAM-1, VCAM-1 can also be shed from the cell membrane into a circulating form (sVCAM-1). Women with severe (grade 3 and 4) endometriosis have been shown to have higher blood levels of sVCAM-1 compared to control patients [88], while other authors have measured increased peritoneal VCAM-1 mRNA in endometriosis patients [29]. Just as VCAM-1 is instrumental in facilitating leukocyte adhesion to the endothelium under inflammatory conditions, it has been shown to play a pivotal role in the adhesion and invasion of tumour cells [89]. The upregulation of VCAM-1 expression on endothelial cells could facilitate the increased adhesion of tumour cells, while its decreased expression in angiogenic vessels might allow tumour cells to escape immunosurveillance via reduced leukocyte infiltration [90]. Clinically, VCAM-1 has been recognised to participate in site-specific metastasis via the spread of breast cancer to the lung [91]. This illustrates the importance of the adhesion molecule–tumour microenvironment in the “seed and soil” hypothesis. For a comprehensive review of the molecular mechanisms of VCAM-1 involvement in malignant invasion and metastasis, interested readers are directed to an excellent review on the topic [92].

Although the evidence suggests a key role for adhesion molecules in endometriosis, it must be balanced against the likelihood of multiple, overlapping pathways functioning in parallel. A key player driving numerous physiological, tumorigenic and immunological processes is the Nuclear Factor Kappa B (NF-κB) family of transcription factors. This pathway has been shown to be activated by numerous physiological mediators and stressors, including inflammatory cytokines, ischaemia, environmental hazards and cigarette smoke [93]. NF-κB is one of the principal regulators of constitutive ICAM-1 and VCAM-1 expression [94]. Therefore, NF-κB unsurprisingly displays considerable influence in the regulation of cellular adhesion molecules in the pathophysiology of endometriosis. For example, when the endometrial samples from normal controls, including normally-located entopic endometrium from endometriosis patients and ectopic (outside of the normal location) endometrium, were analysed for expression of ICAM-1 and NF-κB, the ectopic endometriotic tissue had significantly higher levels of both molecules than entopic endometrium [95]. Higher levels of both NF-κB and ICAM-1 have also been reported in red endometriotic (active inflammatory [96]) lesions as compared to black (inactive) lesions [97]. Furthermore, the inhibition of NF-κB in an animal model of endometriosis reduced ICAM-1 expression and cell proliferation, with endometriotic lesions concurrently undergoing increased apoptosis [98]. In some studies, the changes in ICAM-1 mRNA and sICAM-1 levels were observed even before visible disease, suggesting that ICAM-1 may be involved in the very early pathogenesis of endometriosis as well as its later propagation and deleterious downstream effects [99].

As mentioned previously, endometriosis is known to be a chronic inflammatory state, with immune system dysregulation being a key element of disease persistence. NK cells provide a mechanism for the increased expression of ICAM-1 and VCAM-1 by acting as a source of pro-inflammatory cytokines, such as TNFα and IFN-γ, which signal (at least in part) through the NF-κB pathway. These subsequently increase the cytolytic activity of NK cells via NF-κB-mediated upregulation of ICAM-1 [100,101], demonstrating that ICAM-1 has a key role in the sensitization of cells to lysis by NK cells. 

**Figure 2:**
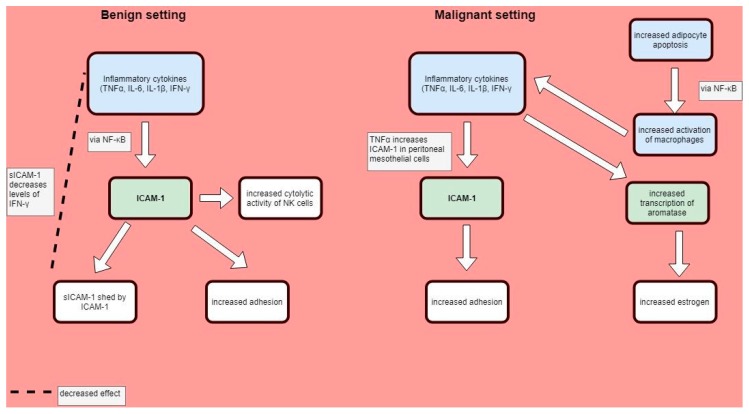
Adhesion molecules in the immune system context.

Conversely, there is significant evidence that supports the hypothesis that NK cell activity is reduced by increased levels of sICAM-1 [102]. Several authors have proposed that insufficient cytotoxic activity against autologous endometrial cells could allow these cells to evade immune surveillance, with resultant clinical dysfunction [103]. Women with endometriosis are indeed noted to have altered peripheral and peritoneal NK cell populations with reduced cytotoxicity. Some authors [76] have proposed an immunologic feedback loop, whereby the increased serum sICAM-1 levels decreased IFN-γ levels in the peritoneal fluid. Furthermore, in this study, there was a significant correlation between the endometrial stromal shedding of soluble ICAM-1 (sICAM-1) and the suppression of NK cell-mediated cytotoxicity, which is consistent with previous work in melanoma [104]. Again, a suggested mechanism is via the inhibition of the ICAM-1/leukocyte function antigen-1 (LFA-1) axis [105], although the possibility of sICAM-1 blocking immune cells from recognising their target is also plausible [106] (Figure 2).

However, this area is controversial in light of evidence that an increased inflammatory cytokine environment (IL-6, TGF-β) *suppresses* NK cytotoxic activity [107], allowing endometriotic lesions to escape peritoneal surveillance and chronic inflammation to continue. This serves to illustrate the complex interplay between the immune system and the microenvironment, with the possibility that the same molecular pathways could be activated or suppressed depending upon specific spatial and temporal contexts.

Furthermore, this pathway may be bidirectional as women with endometriosis have been shown to have a greater proportion of immature NK cells and the surgical resection of endometriotic lesions increases the proportion of mature NK cells [108]. Interestingly, the correlations have been observed between the endometrial supernatant concentration of sICAM-1 and the percentage of NK-mediated lysis inhibited in these samples [67]. In the same population, it was observed that the sICAM-1 formation was significantly higher in patients with advanced disease, lending further weight to the hypothesis that the evasion of immunosurveillance in endometriosis is partially mediated through a sICAM-1 pathway. Indeed, the investigations in patients with advanced gastric cancer (as compared to healthy controls) have shown increased metastatic behaviour and reduced NK functioning in parallel with increased sICAM-1 levels [109]. Similarly, the serum sICAM-1 levels have been related to both tumour stage and loss of cellular cytotoxicity in colorectal cancer [106], which has been proposed as an unfavourable prognostic marker in melanoma [110]. Although it is not currently known whether sICAM-1 influences NK cell function, one report has suggested sICAM-1 as a growth factor for NK cells [111]. Future investigations may shed further light on whether this is diagnostically or clinically important.

## 5. Malignancy

Some crossover is to be expected between ectopic endometrial implantation/invasion pathways and the pathways of invasion in malignant disease. After all, endometriosis involves the spread of tissue from its origin and implantation at a distant site with invasion of local structures. Indeed, there is evidence of a greater magnitude of effect in endometriosis, which is a “benign” disease, than that in malignant melanoma in terms of both sICAM-1 release and cell cytotoxicity suppression [112]. As mentioned, there is an uncommon but well established increased risk of gynaecological malignancy in the patients with endometriosis, which involve mostly ovarian endometrioid adeno- and clear cell carcinomas [113]. It should be noted that these data are epidemiological rather than directly causal [114], although there are several putative mechanisms relevant to this discussion. Trauma and resultant inflammation has been shown to induce endometriosis in hysterotomy scars in animals [115] and women [116], with rare cases of the transformation of scar endometriosis progressing to frank malignancy [117]. Although it is likely that multiple parallel and convergent molecular signalling pathways are involved, one player that has received attention is the tumour suppressor gene phosphatase and tensin homolog (PTEN). Mutations in PTEN, which normally encodes a phosphatase that acts as a negative regulator of the pro-tumorigenic phosphoinositide (PI) 3-kinase signalling pathway, have been implicated in the progression from endometrial cysts to endometrioid ovarian carcinoma. Specifically, certain PTEN mutations have been described in the early events in the development of abnormal endometrial hyperplasia [118] in addition to being well described in endometrioid endometrial cancer [119]. This pathway is of note given the role of PTEN in suppressing cell adhesion and migration [120]. By dephosphorylating the tyrosine kinase focal adhesion kinase (FAK) among other potential targets, PTEN has the potential to reduce FAK-mediated downstream integrin signalling pathways [121]. Since FAK dysregulation has been implicated in many cancers [122], this serves to underline the complexity of integrin activation signalling within tumour subtypes and their individual microenvironments.

In terms of adhesion–immune interactions, the role of a reactive tumour stroma, comprised of a heterogenous cellular milieu actively promoting angiogenesis, inflammation, immune evasion and growth, has been well described [123]. This interaction in a hormonal tissue microenvironment is best recognised in breast cancer, but it is reasonable to speculate that this also could have considerable implications for the endometrial setting. In particular, the role of tumour-associated macrophages (TAMs) has been noted to have a crucial role in metastatic tumour behaviour in breast cancer, with the innate properties of the immune system essentially being subverted to serve the purposes of an invasive mass [124]. The functional interactions between macrophages and inflammatory cytokines are of major clinical importance in breast cancer, whereby the increased adipocyte apoptosis and release of free fatty acids has been observed to activate the NF-κB pathway. In turn, this activates macrophages, which subsequently secrete pro-inflammatory factors, including TNF-α, IL-1β, IL-6 and PgE2. The increased production of the inflammatory factors stimulates the transcription of aromatase, which is the rate-limiting step in oestrogen production [125] (see Figure 2). Similarly, high circulating oestrogen levels arising from the peripheral conversion of androgens in overweight women increases the risk of endometrial cancer, with a direct relationship between BMI and increasing risk [126]. The rate of endometrial cancer has increased in line with the increased prevalence of obesity [127] and although unopposed oestrogen stimulation of the endometrium is the main risk factor for endometrial cancer, obesity has been demonstrated to be an oestrogen-independent risk factor [128].

In breast cancer, the expression of hormone receptors on tumours plays an integral role in determining both tumour character and accordingly patient prognosis, with tumours responding to anti-hormonal agents having a significantly more favourable outlook than those that are hormone receptor negative [129,130]. Oestrogen has been proposed to inhibit both inducible and constitutive activation of NF-κB, with an implication that breast cancers lacking a functional oestrogen receptor (ER) have higher NF-κB activity and thus, overexpress key genes that might promote tumorigenesis [131]. As discussed, NF-κB regulates the expression of several adhesion molecules, including ICAM-1 and VCAM-1, illustrating one potential pathway whereby the adhesion molecules may be influenced by the hormonal milieu. Likewise, the adhesion and proteolytic mediators are influenced by the hormonal environment and in fact, this bidirectional relationship has been a focus of study for many years. For example, increased MMP-2 activation has been noted in established hormone receptor-negative cell lines, while the MMP expression has been shown to be elevated in general as breast cancer cells progress to an oestrogen-resistant phenotype in vitro [132].

Indeed, the cooperation between adhesive and proteolytic processes is intuitively of great importance in invasive cellular behaviour, especially in light of the demonstrated involvement of sICAM-1 and sVCAM-1 in the pathophysiology of endometriosis. Integrins, such as αVβ3, have been shown to co-localise with matrix metalloproteinases at the invasive front of tumour cells to facilitate angiogenesis in cultured melanoma cells in vivo [133]. It has been proposed that the spatial co-localisation of NF-kB with the transcription factor activating protein 1 (AP-1) increases MMP expression in HeLa and mammary carcinoma cell lines [134]. Matrix metalloprotease (MMP)-mediated cleavage of ICAM-1 has also been proposed as an actor in the NF-kB pathway. Specifically, under the influence of cytokines, such as TNF, NF-kB is released from its inhibitory cytoplasmic protein IκB and translocates to the nucleus to facilitate the activation of matrix metalloproteases [135]. Indeed, ICAM-1 is a target for drug nanocarriers, with the inhibition of MMPs having a secondary, somewhat unintended, benefit of reducing sICAM-1 release [136]. In a similar fashion, the activation of endothelial NADPH by VCAM-1 releases reactive oxygen species. These reactive oxygen species are crucial in VCAM-1 mediated lymphocyte migration, but also in activation of matrix metalloproteinases 2 and 9 [137]. In a similar manner, the soluble form of VCAM is shed via the activity of ADAM enzymes [138].

The role of NF-kB mediation of cellular adhesion processes is also of interest in the context of cancer immunotherapy [139], although the mechanism by which pathological NF-kB activation occurs in malignant disease remains a subject of ongoing debate. The role of decoy receptor 3 (DcR3) has been proposed as a potential mechanism to promote cell adhesion in the development of endometriosis [140]. DcR3 has long been recognised as a method by which cancer cells may evade NK and cytotoxic T-cells via the inhibition of FasL-induced apoptosis [141]. Tsai et al. [140] noted an increased activation of the Akt-NF-kB signalling pathway in ectopic endometrium, with consequent upregulation of DcR3. In this xenograft study, DcR3 expression levels correlated both with ICAM-1 levels and with those of homing cell adhesion molecule (HCAM). A knockdown of DcR3 was found to reduce cell adhesion and migration. It should be further noted that DcR3 is regulated by oestrogen and indeed, DcR3 knockdown have been shown to reduce invasive phenotypes in the breast cancer cell line MCF-7 [142]. It is evident that the various adhesion molecules have multi-fold complex actions in the malignant setting and in “benign” diseases. The greater understanding of these processes holds considerable potential in the development of biomarkers and therapeutics.

## 6. Clinical Applications

Although huge strides have undoubtedly been made in cancer treatments, there are still many areas in which the clinicians are fundamentally at a loss to explain why one patient may respond well to a therapy and another does not, even though they may share identical tumour molecular signatures. Some examples of this are that not all HER2-positive breast cancer patients respond well to anti-HER2 therapies; or in the case of “benign” proliferative conditions, patients with similar diagnoses often exhibit variable responses to hormonal treatments. A deeper understanding of the molecular mechanisms of dysregulated adhesion has suggested some promising therapeutic targets that may not be intuitively obvious, such as the use of an anti-cholesterol drug, simvastatin, to reduce ICAM-1, VCAM-1 and integrin α4β1 expression on the tumour cells invading the peritoneum [143]. Similarly, the inhibition of the NF-κB pathway in the tumour microenvironment has shown some promise [139] although this may prove best in conjunction with the existing therapies. Although soluble adhesion molecules have been shown to be elevated in diverse malignancies, such as breast, lung, gastrointestinal, ovarian and some haematological cancers [144,145,146,147], considerable work remains to be done before the biomarkers or drugs based on these molecules can be applied in standard oncological care.

Similarly, although the pathways outlined in this review offer at least a partial mechanistic explanation for the invasive pathophysiology of endometriosis, there are multiple confounders, such as prior medical treatment, surgical resection, individual patient factors etc. The search to find a suitable endometriosis biomarker in peripheral blood is urgent, as invasive laparoscopy, an expensive procedure that is not without risk or complications, continues to be the gold standard [148]. Furthermore, the current practice is such that the time to diagnosis is measured in years rather than months [149]. However, a recent Cochrane review of proposed biomarkers of endometriosis, including adhesion molecules, noted that the evidence was not yet strong enough to recommend any particular marker, with studies being generally small and sometimes conflicting. For example, in the case of sICAM-1, the inconsistent results comparing endometriosis patients with controls may be due to a variety of factors, including previous treatments and the timing of menstrual cycles [150]. Given the complexities of the adhesive microenvironment, it is unsurprising that robust directly linear correlations are difficult to make [77]. However, it is hoped that the continued investigation into the interaction of adhesion molecules with their microenvironment will yield novel and exploitable insights into the disease processes and treatment in the future.

## References

[1] Mukherjee S. (2017). Cancer’s Invasion Equation. The New Yorker.

[2] Bosch-Fortea M., Martín-Belmonte F. (2018). Mechanosensitive Adhesion Complexes in Epithelial Architecture and Cancer Onset. Curr. Opin. Cell Biol..

[3] Ladoux B., Mège R.-M. (2017). Mechanobiology of Collective Cell Behaviours. Nat. Rev. Mol. Cell Biol..

[4] Ford A.J., Rajagopalan P. (2017). Extracellular Matrix Remodeling in 3D: Implications in Tissue Homeostasis and Disease Progression. Wiley Interdiscip. Rev. Nanomed. Nanobiotechnol..

[5] Ebnet K. (2017). Junctional Adhesion Molecules JAMs): Cell Adhesion Receptors With Pleiotropic Functions in Cell Physiology and Development. Physiol. Rev..

[6] Paget S. (1989). The Distribution of Secondary Growths in Cancer of the Breast. 1889. Cancer Metastasis Rev..

[7] Bland K.I., Klimberg V.S., Copeland E.M. (2018). 30 - Halsted Radical Mastectomy. The Breast.

[8] Yanaihara N., Caplen N., Bowman E., Seike M., Kumamoto K., Yi M., Stephens R.M., Okamoto A., Yokota J., Tanaka T. (2006). Unique microRNA Molecular Profiles in Lung Cancer Diagnosis and Prognosis. Cancer Cell.

[9] Wei S., Siegal G.P. (2018). Surviving at a Distant Site: The Organotropism of Metastatic Breast Cancer. Semin. Diagn. Pathol..

[10] Hart I.R., Fidler I.J. (1980). Role of Organ Selectivity in the Determination of Metastatic Patterns of B16 Melanoma. Cancer Res..

[11] Fidler I.J. (2003). The Pathogenesis of Cancer Metastasis: The “Seed and Soil” Hypothesis Revisited. Nat. Rev. Cancer.

[12] Agarwal N., Subramanian A. (2010). Endometriosis - Morphology, Clinical Presentations and Molecular Pathology. J. Lab. Physicians.

[13] Sampson J.A. (1927). Peritoneal Endometriosis due to the Menstrual Dissemination of Endometrial Tissue into the Peritoneal Cavity. Am. J. Obstet. Gynecol..

[14] Bulun S.E. (2009). Endometriosis. N. Engl. J. Med..

[15] Jess T., Frisch M., Jørgensen K.T., Pedersen B.V., Nielsen N.M. (2012). Increased Risk of Inflammatory Bowel Disease in Women with Endometriosis: A Nationwide Danish Cohort Study. Gut.

[16] Yazdani A. (2018). Right of Reply to: Surgical Treatment Is an Excellent Option for Women with Endometriosis and Infertility. Aust. N. Z. J. Obstet. Gynaecol..

[17] Hynes R.O. (1987). Integrins: A Family of Cell Surface Receptors. Cell.

[18] Changede R., Sheetz M. (2017). Integrin and Cadherin Clusters: A Robust Way to Organize Adhesions for Cell Mechanics. Bioessays.

[19] Kim C., Ye F., Ginsberg M.H. (2011). Regulation of Integrin Activation. Annu. Rev. Cell Dev. Biol..

[20] Gahmberg C.G., Fagerholm S.C., Nurmi S.M., Chavakis T., Marchesan S., Grönholm M. (2009). Regulation of Integrin Activity and Signalling. Biochim. Biophys. Acta.

[21] Ginsberg M.H., Partridge A., Shattil S.J. (2005). Integrin Regulation. Curr. Opin. Cell Biol..

[22] Danen E.H.J. (2013). Integrins: An Overview of Structural and Functional Aspects.

[23] Ruck P., Marzusch K., Kaiserling E., Horny H.P., Dietl J., Geiselhart A., Handgretinger R., Redman C.W. (1994). Distribution of Cell Adhesion Molecules in Decidua of Early Human Pregnancy. An Immunohistochemical Study. Lab. Investig..

[24] Lessey B.A., Damjanovich L., Coutifaris C., Castelbaum A., Albelda S.M., Buck C.A. (1992). Integrin Adhesion Molecules in the Human Endometrium. Correlation with the Normal and Abnormal Menstrual Cycle. J. Clin. Investig..

[25] Tavaniotou A., Bourgain C., Albano C., Platteau P., Smitz J., Devroey P. (2003). Endometrial Integrin Expression in the Early Luteal Phase in Natural and Stimulated Cycles for in Vitro Fertilization. Eur. J. Obstet. Gynecol. Reprod. Biol..

[26] Lessey B.A. (1998). Endometrial Integrins and the Establishment of Uterine Receptivity. Hum. Reprod..

[27] Lin X., Dai Y., Xu W., Shi L., Jin X., Li C., Zhou F., Pan Y., Zhang Y., Lin X. (2018). Hypoxia Promotes Ectopic Adhesion Ability of Endometrial Stromal Cells via TGF-β1/Smad Signalling in Endometriosis. Endocrinology.

[28] Choi H.-J., Park M.-J., Kim B.-S., Choi H.-J., Joo B., Lee K.S., Choi J.-H., Chung T.-W., Ha K.-T. (2017). Transforming Growth Factor β1 Enhances Adhesion of Endometrial Cells to Mesothelium by Regulating Integrin Expression. BMB Rep..

[29] Kyama C.M., Overbergh L., Mihalyi A., Meuleman C., Mwenda J.M., Mathieu C., D’Hooghe T.M. (2008). Endometrial and Peritoneal Expression of Aromatase, Cytokines, and Adhesion Factors in Women with Endometriosis. Fertil. Steril..

[30] Charo I.F., Nannizzi L., Smith J.W., Cheresh D.A. (1990). The Vitronectin Receptor Alpha v Beta 3 Binds Fibronectin and Acts in Concert with Alpha 5 Beta 1 in Promoting Cellular Attachment and Spreading on Fibronectin. J. Cell Biol..

[31] Béliard A., Donnez J., Nisolle M., Foidart J.M. (1997). Localization of Laminin, Fibronectin, E-Cadherin, and Integrins in Endometrium and Endometriosis. Fertil. Steril..

[32] Ahmad S.F., Brown J.K., Campbell L.L., Koscielniak M., Oliver C., Wheelhouse N., Entrican G., McFee S., Wills G.S., McClure M.O. (2018). Pelvic Chlamydial Infection Predisposes to Ectopic Pregnancy by Upregulating Integrin β1 to Promote Embryo-Tubal Attachment. EBioMedicine.

[33] Sillem M., Prifti S., Monga B., Arslic T., Runnebaum B. (1999). Integrin-Mediated Adhesion of Uterine Endometrial Cells from Endometriosis Patients to Extracellular Matrix Proteins Is Enhanced by Tumor Necrosis Factor Alpha TNFαand Interleukin-1 IL-1). Eur. J. Obstet. Gynecol. Reprod. Biol..

[34] Daftary G.S., Troy P.J., Bagot C.N., Young S.L., Taylor H.S. (2002). Direct Regulation of beta3-Integrin Subunit Gene Expression by HOXA10 in Endometrial Cells. Mol. Endocrinol..

[35] Kitawaki J., Harada T. (2014). Sex Steroids and Endometriosis. Endometriosis: Pathogenesis and Treatment.

[36] Lessey B.A., Castelbaum A.J., Sawin S.W., Buck C.A., Schinnar R., Bilker W., Strom B.L. (1994). Aberrant Integrin Expression in the Endometrium of Women with Endometriosis. J. Clin. Endocrinol. Metab..

[37] Elnaggar A., Farag A.H., Gaber M.E., Hafeez M.A., Ali M.S., Atef A.M. (2017). AlphaVBeta3 Integrin Expression within Uterine Endometrium in Unexplained Infertility: A Prospective Cohort Study. BMC Womens. Health.

[38] Bildirici I., Bukulmez O., Ensari A., Yarali H., Gurgan T. (2001). A Prospective Evaluation of the Effect of Salpingectomy on Endometrial Receptivity in Cases of Women with Communicating Hydrosalpinges. Hum. Reprod..

[39] Johnson N., van Voorst S., Sowter M.C., Strandell A., Mol B.W.J. (2010). Surgical Treatment for Tubal Disease in Women due to Undergo in Vitro Fertilisation. Cochrane Database Syst. Rev..

[40] Regidor P.A., Vogel C., Regidor M., Schindler A.E., Winterhager E. (1998). Expression Pattern of Integrin Adhesion Molecules in Endometriosis and Human Endometrium. Hum. Reprod. Update.

[41] Rakhila H., Girard K., Leboeuf M., Lemyre M., Akoum A. (2014). Macrophage Migration Inhibitory Factor Is Involved in Ectopic Endometrial Tissue Growth and Peritoneal-Endometrial Tissue Interaction in Vivo: A Plausible Link to Endometriosis Development. PLoS ONE.

[42] Bondza P.K., Metz C.N., Akoum A. (2008). Macrophage Migration Inhibitory Factor up-Regulates alpha(v)beta(3) Integrin and Vascular Endothelial Growth Factor Expression in Endometrial Adenocarcinoma Cell Line Ishikawa. J. Reprod. Immunol..

[43] Lindsay S.F., Luciano D.E., Luciano A.A. (2015). Emerging Therapy for Endometriosis. Expert Opin. Emerg. Drugs.

[44] Franke H.R., van de Weijer P.H., Pennings T.M., van der Mooren M.J. (2000). Gonadotropin-Releasing Hormone Agonist plus “Add-Back” Hormone Replacement Therapy for Treatment of Endometriosis: A Prospective, Randomized, Placebo-Controlled, Double-Blind Trial. Fertil. Steril..

[45] Groulx J.-F., Boudjadi S., Beaulieu J.-F., Civera M., Arosio D., Bonato F., Manzoni L., Pignataro L., Zanella S., Gennari C. Special Issue “Integrins in Cancer”, Cancers. http://www.mdpi.com/journal/cancers/special_issues/integrin.

[46] Lochter A., Navre M., Werb Z., Bissell M.J. (1999). alpha1 and alpha2 Integrins Mediate Invasive Activity of Mouse Mammary Carcinoma Cells through Regulation of Stromelysin-1 Expression. Mol. Biol. Cell.

[47] Desgrosellier J.S., Cheresh D.A. (2010). Integrins in Cancer: Biological Implications and Therapeutic Opportunities. Nat. Rev. Cancer.

[48] Witkowska A.M., Borawska M.H. (2004). Soluble Intercellular Adhesion Molecule-1 sICAM-1): An Overview. Eur. Cytokine Netw..

[49] Bella J., Kolatkar P.R., Marlor C.W., Greve J.M., Rossmann M.G. (1998). The Structure of the Two Amino-Terminal Domains of Human ICAM-1 Suggests How It Functions as a Rhinovirus Receptor and as an LFA-1 Integrin Ligand. Proc. Natl. Acad. Sci. USA.

[50] Rothlein R., Dustin M.L., Marlin S.D., Springer T.A. (1986). A Human Intercellular Adhesion Molecule ICAM-1Distinct from LFA-1. J. Immunol..

[51] Newham P., Craig S.E., Seddon G.N., Schofield N.R., Rees A., Edwards R.M., Jones E.Y., Humphries M.J. (1997). α4 Integrin Binding Interfaces on VCAM-1 and MAdCAM-1: Integrin Binding Footprints Identify Accessory Binding Sites That Play a Role in Integrin Specificity. J. Biol. Chem..

[52] Rose D.M., Cardarelli P.M., Cobb R.R., Ginsberg M.H. (2000). Soluble VCAM-1 Binding to alpha4 Integrins Is Cell-Type Specific and Activation Dependent and Is Disrupted during Apoptosis in T Cells. Blood.

[53] Clements J.M., Newham P., Shepherd M., Gilbert R., Dudgeon T.J., Needham L.A., Edwards R.M., Berry L., Brass A., Humphries M.J. (1994). Identification of a Key Integrin-Binding Sequence in VCAM-1 Homologous to the LDV Active Site in Fibronectin. J. Cell Sci..

[54] Springer T.A. (1994). Traffic Signals for Lymphocyte Recirculation and Leukocyte Emigration: The Multistep Paradigm. Cell.

[55] Tawia S.A., Beaton L.A., Rogers P.A. (1993). Immunolocalization of the Cellular Adhesion Molecules, Intercellular Adhesion Molecule-1 ICAM-1and Platelet Endothelial Cell Adhesion Molecule PECAM), in Human Endometrium throughout the Menstrual Cycle. Hum. Reprod..

[56] Thomson A.J., Greer M.R., Young A., Boswell F., Telfer J.F., Cameron I.T., Norman J.E., Campbell S. (1999). Expression of Intercellular Adhesion Molecules ICAM-1 and ICAM-2 in Human Endometrium: Regulation by Interferon-Gamma. Mol. Hum. Reprod..

[57] Seaward A.V.C., Burke S.D., Croy B.A. (2010). Interferon Gamma Contributes to Preimplantation Embryonic Development and to Implantation Site Structure in NOD Mice. Hum. Reprod..

[58] Bevilacqua M.P., Nelson R.M., Mannori G., Cecconi O. (1994). Endothelial-Leukocyte Adhesion Molecules in Human Disease. Annu. Rev. Med..

[59] Albelda S.M., Smith C.W., Ward P.A. (1994). Adhesion Molecules and Inflammatory Injury. FASEB J..

[60] Yusuf-Makagiansar H., Anderson M.E., Yakovleva T.V., Murray J.S., Siahaan T.J. (2002). Inhibition of LFA-1/ICAM-1 and VLA-4/VCAM-1 as a Therapeutic Approach to Inflammation and Autoimmune Diseases. Med. Res. Rev..

[61] Milewski Ł., Dziunycz P., Barcz E., Radomski D., Roszkowski P.I., Korczak-Kowalska G., Kamiński P., Malejczyk J. (2011). Increased Levels of Human Neutrophil Peptides 1, 2, and 3 in Peritoneal Fluid of Patients with Endometriosis: Association with Neutrophils, T Cells and IL-8. J. Reprod. Immunol..

[62] Koninckx P.R., Kennedy S.H., Barlow D.H. (1998). Endometriotic Disease: The Role of Peritoneal Fluid. Hum. Reprod. Update.

[63] Beliard A., Noël A., Goffin F., Frankenne F., Foidart J.-M. Adhesion of Endometrial Cells Labeled with 111lndium-Tropolonate to Peritoneum: A Novel in Vitro Model to Study Endometriosis. https://orbi.uliege.be/bitstream/2268/12348/1/B%c3%a9liard%20A%20FS%202003.pdf.pdf.

[64] Shiga K., Hara M., Nagasaki T., Sato T., Takahashi H., Takeyama H. (2015). Cancer-Associated Fibroblasts: Their Characteristics and Their Roles in Tumor Growth. Cancers.

[65] Alkhamesi N.A., Ziprin P., Pfistermuller K., Peck D.H., Darzi A.W. (2005). ICAM-1 Mediated Peritoneal Carcinomatosis, a Target for Therapeutic Intervention. Clin. Exp. Metastasis.

[66] Ziprin P., Ridgway P.F., Pfistermüller K.L.M., Peck D.H., Darzi A.W. (2003). ICAM-1 Mediated Tumor-Mesothelial Cell Adhesion Is Modulated by IL-6 and TNF-Alpha: A Potential Mechanism by Which Surgical Trauma Increases Peritoneal Metastases. Cell Commun. Adhes..

[67] Somigliana E., Viganò P., Gaffuri B., Guarneri D., Busacca M., Vignali M. (1996). Human Endometrial Stromal Cells as a Source of Soluble Intercellular Adhesion Molecule ICAM)-1 Molecules. Hum. Reprod..

[68] Glowinska B., Urban M., Peczynska J., Florys B. (2005). Soluble Adhesion Molecules sICAM-1, sVCAM-1and Selectins sE Selectin, sP Selectin, sL SelectinLevels in Children and Adolescents with Obesity, Hypertension, and Diabetes. Metabolism.

[69] Marguet C., Dean T.P., Warner J.O. (2000). Soluble Intercellular Adhesion Molecule-1 sICAM-1and Interferon-Gamma in Bronchoalveolar Lavage Fluid from Children with Airway Diseases. Am. J. Respir. Crit. Care Med..

[70] Keaney J.F., Massaro J.M., Larson M.G., Vasan R.S., Wilson P.W.F., Lipinska I., Corey D., Sutherland P., Vita J.A., Benjamin E.J. (2004). Heritability and Correlates of Intercellular Adhesion Molecule-1 in the Framingham Offspring Study. J. Am. Coll. Cardiol..

[71] Ballantyne C.M., Entman M.L. (2002). Soluble Adhesion Molecules and the Search for Biomarkers for Atherosclerosis. Circulation.

[72] Somigliana E., Viganò P., Candiani M., Felicetta I., Di Blasio A.M., Vignali M. (2002). Use of Serum-Soluble Intercellular Adhesion Molecule-1 as a New Marker of Endometriosis. Fertil. Steril..

[73] Kuessel L., Wenzl R., Proestling K., Balendran S., Pateisky P., Yotova, Yerlikaya G., Streubel B., Husslein H. (2017). Soluble VCAM-1/soluble ICAM-1 Ratio Is a Promising Biomarker for Diagnosing Endometriosis. Hum. Reprod..

[74] Rzymski P., Woźniak J., Opala T. (2003). [Production of soluble intracellular adhesion molecule-1 sICAM-1in human endometrial cell culture]. Wiad. Lek..

[75] Viganò P., Somigliana E., Gaffuri B., Santorsola R., Busacca M., Vignali M. (2000). Endometrial Release of Soluble Intercellular Adhesion Molecule 1 and Endometriosis: Relationship to the Extent of the Disease. Obstet. Gynecol..

[76] Wu M.H., Yang B.C., Hsu C.C., Lee Y.C., Huang K.E. (1998). The Expression of Soluble Intercellular Adhesion Molecule-1 in Endometriosis. Fertil. Steril..

[77] May K.E., Conduit-Hulbert S.A., Villar J., Kirtley S., Kennedy S.H., Becker C.M. (2010). Peripheral Biomarkers of Endometriosis: A Systematic Review. Hum. Reprod. Update.

[78] Steff A.-M., Gagné D., Pagé M., Hugo P., Gosselin D. (2004). Concentration of Soluble Intercellular Adhesion Molecule-1 in Serum Samples from Patients with Endometriosis Collected during the Luteal Phase of the Menstrual Cycle. Hum. Reprod..

[79] Daniel Y., Geva E., Amit A., Eshed-Englender T., Baram A., Fait G., Lessing J.B. (2000). Do Soluble Cell Adhesion Molecules Play a Role in Endometriosis?. Am. J. Reprod. Immunol..

[80] Fonsatti E., Altomonte M., Coral S., Cattarossi I., Nicotra M.R., Gasparollo A., Natali P.G., Maio M. (1997). Tumour-Derived Interleukin 1alpha IL-1alphaup-Regulates the Release of Soluble Intercellular Adhesion Molecule-1 sICAM-1by Endothelial Cells. Br. J. Cancer.

[81] Osborn L., Hession C., Tizard R., Vassallo C., Luhowskyj S., Chi-Rosso G., Lobb R. (1989). Direct Expression Cloning of Vascular Cell Adhesion Molecule 1, a Cytokine-Induced Endothelial Protein That Binds to Lymphocytes. Cell.

[82] Freedman A.S., Munro J.M., Rice G.E., Bevilacqua M.P., Morimoto C., McIntyre B.W., Rhynhart K., Pober J.S., Nadler L.M. (1990). Adhesion of Human B Cells to Germinal Centers in Vitro Involves VLA-4 and INCAM-110. Science.

[83] Barreiro O., Yáñez-Mó M., Sala-Valdés M., Gutiérrez-López M.D., Ovalle S., Higginbottom A., Monk P.N., Cabañas C., Sánchez-Madrid F. (2005). Endothelial Tetraspanin Microdomains Regulate Leukocyte Firm Adhesion during Extravasation. Blood.

[84] Imhof B.A., Dunon D., Dixon F.J. (1995). Leukocyte Migration and Adhesion. Advances in Immunology.

[85] Cook-Mills J.M., Marchese M.E., Abdala-Valencia H. (2011). Vascular Cell Adhesion Molecule-1 Expression and Signaling during Disease: Regulation by Reactive Oxygen Species and Antioxidants. Antioxid. Redox Signal..

[86] Tabibzadeh S., Kong Q.F., Babaknia A. (1994). Expression of Adhesion Molecules in Human Endometrial Vasculature throughout the Menstrual Cycle. J. Clin. Endocrinol. Metab..

[87] Schutt A.K., Atkins K.A., Slack-Davis J.K., Stovall D.W. (2015). VCAM-1 on Peritoneum and α4β1 Integrin in Endometrium and Their Implications in Endometriosis. Int. J. Gynecol. Pathol..

[88] Barrier B.F., Sharpe-Timms K.L. (2002). Expression of Soluble Adhesion Molecules in Sera of Women with Stage III and IV Endometriosis. J. Soc. Gynecol. Investig..

[89] Martin-Padura I., Mortarini R., Lauri D., Bernasconi S., Sanchez-Madrid F., Parmiani G., Mantovani A., Anichini A., Dejana E. (1991). Heterogeneity in Human Melanoma Cell Adhesion to Cytokine Activated Endothelial Cells Correlates with VLA-4 Expression1. Cancer Res..

[90] Griffioen A.W., Damen C.A., Martinotti S., Blijham G.H., Groenewegen G. (1996). Endothelial Intercellular Adhesion Molecule-1 Expression Is Suppressed in Human Malignancies: The Role of Angiogenic Factors. Cancer Res..

[91] Minn A.J., Gupta G.P., Siegel P.M., Bos P.D., Shu W., Giri D.D., Viale A., Olshen A.B., Gerald W.L., Massagué J. (2005). Genes That Mediate Breast Cancer Metastasis to Lung. Nature.

[92] Schlesinger M., Bendas G. (2015). Vascular Cell Adhesion Molecule-1 VCAM-1)--an Increasing Insight into Its Role in Tumorigenicity and Metastasis. Int. J. Cancer.

[93] Sen R., Baltimore D. (1986). Inducibility of Kappa Immunoglobulin Enhancer-Binding Protein Nf-Kappa B by a Posttranslational Mechanism. Cell.

[94] Xia Y.F., Liu L.P., Zhong C.P., Geng J.G. (2001). NF-kappaB Activation for Constitutive Expression of VCAM-1 and ICAM-1 on B Lymphocytes and Plasma Cells. Biochem. Biophys. Res. Commun..

[95] Wang F., He Y.-L., Peng D.-X., Liu M.-B. (2005). Expressions of nuclear factor-kappaB and intercellular adhesion molecule-1 in endometriosis. Di Yi Jun Yi Da Xue Xue Bao.

[96] Khan K.N., Kitajima M., Hiraki K., Fujishita A., Nakashima M., Masuzaki H. (2014). Visible and Occult Microscopic Lesions of Endometriosis. Gynecol. Minim. Invasive Ther..

[97] González-Ramos R., Donnez J., Defrère S., Leclercq I., Squifflet J., Lousse J.-C., Van Langendonckt A. (2007). Nuclear Factor-Kappa B Is Constitutively Activated in Peritoneal Endometriosis. Mol. Hum. Reprod..

[98] González-Ramos R., van Langendonckt A., Defrère S., Lousse J.-C., Colette S., Devoto L., Donnez J. (2010). Involvement of the Nuclear Factor-κB Pathway in the Pathogenesis of Endometriosis. Fertil. Steril..

[99] Wu M.-H., Yang B.-C., Lee Y.-C., Wu P.-L., Hsu C.-C. (2004). The Differential Expression of Intercellular Adhesion Molecule-1 ICAM-1and Regulation by Interferon-Gamma during the Pathogenesis of Endometriosis. Am. J. Reprod. Immunol..

[100] Wang R., Jaw J.J., Stutzman N.C., Zou Z., Sun P.D. (2012). Natural Killer Cell-Produced IFN-γ and TNF-α Induce Target Cell Cytolysis through up-Regulation of ICAM-1. J. Leukoc. Biol..

[101] Defrère S., Donnez J., Moulin P., Befahy P., Gonzalez-Ramos R., Lousse J.-C., Van Langendonckt A. (2008). Expression of Intercellular Adhesion Molecule-1 and Vascular Cell Adhesion Molecule-1 in Human Endometrial Stromal and Epithelial Cells Is Regulated by Interferon-Gamma but Not Iron. Gynecol. Obstet. Investig..

[102] Fukaya T., Sugawara J., Yoshida H., Murakami T., Yajima A. (1999). Intercellular Adhesion Molecule-1 and Hepatocyte Growth Factor in Human Endometriosis: Original Investigation and a Review of Literature. Gynecol. Obstet. Investig..

[103] Viganò P., Vercellini P., Di Blasio A.M., Colombo A., Candiani G.B., Vignali M. (1991). Deficient Antiendometrium Lymphocyte-Mediated Cytotoxicity in Patients with Endometriosis. Fertil. Steril..

[104] Altomonte M., Gloghini A., Bertola G., Gasparollo A., Carbone A., Ferrone S., Maio M. (1993). Differential Expression of Cell Adhesion Molecules CD54/CD11a and CD58/CD2 by Human Melanoma Cells and Functional Role in Their Interaction with Cytotoxic Cells. Cancer Res..

[105] Viganó P., Pardi R., Magri B., Busacca M., Di Blasio A.M., Vignali M. (1994). Expression of Intercellular Adhesion Molecule-1 ICAM-1on Cultured Human Endometrial Stromal Cells and Its Role in the Interaction with Natural Killers. Am. J. Reprod. Immunol..

[106] Sánchez-Rovira P., Jimenez E., Carracedo J., Barneto I.C., Ramirez R., Aranda E. (1998). Serum Levels of Intercellular Adhesion Molecule 1 ICAM-1in Patients with Colorectal Cancer: Inhibitory Effect on Cytotoxicity. Eur. J. Cancer.

[107] Izumi G., Koga K., Takamura M., Makabe T., Satake E., Takeuchi A., Taguchi A., Urata Y., Fujii T., Osuga Y. (2018). Involvement of Immune Cells in the Pathogenesis of Endometriosis. J. Obstet. Gynaecol. Res..

[108] Thiruchelvam U., Wingfield M., O’Farrelly C. (2015). Natural Killer Cells: Key Players in Endometriosis. Am. J. Reprod. Immunol..

[109] Kaihara A., Iwagaki H., Gouchi A., Hizuta A., Isozaki H., Takakura N., Tanaka N. (1998). Soluble Intercellular Adhesion Molecule-1 and Natural Killer Cell Activity in Gastric Cancer Patients. Res. Commun. Mol. Pathol. Pharmacol..

[110] Altomonte M., Colizzi F., Esposito G., Maio M. (1992). Circulating Intercellular Adhesion Molecule 1 as a Marker of Disease Progression in Cutaneous Melanoma. N. Engl. J. Med..

[111] Takahara M., Nagato T., Komabayashi Y., Yoshino K., Ueda S., Kishibe K., Harabuchi Y. (2013). Soluble ICAM-1 Secretion and Its Functional Role as an Autocrine Growth Factor in Nasal NK/T Cell Lymphoma Cells. Exp. Hematol..

[112] Viganò P., Somigliana E., Di Blasio A.M., Cozzolino S., Candiani M., Vignali M. (2001). Suppression of Natural Killer Cell Function and Production of Soluble ICAM-1: Endometrial Stroma versus Melanoma. Am. J. Reprod. Immunol..

[113] Heaps J.M., Nieberg R.K., Berek J.S. (1990). Malignant Neoplasms Arising in Endometriosis. Obstet. Gynecol..

[114] Krawczyk N., Banys-Paluchowski M., Schmidt D., Ulrich U., Fehm T. (2016). Endometriosis-Associated Malignancy. Geburtshilfe Frauenheilkd..

[115] Hadfield R.M., Yudkin P.L., Coe C.L., Scheffler J., Uno H., Barlow D.H., Kemnitz J.W., Kennedy S.H. (1997). Risk Factors for Endometriosis in the Rhesus Monkey Macaca Mulatta): A Case-Control Study. Hum. Reprod. Update.

[116] Malutan A.M., Simon I., Ciortea R., Mocan-Hognogi R.F., Dudea M., Mihu D. (2017). Surgical Scar Endometriosis: A Series of 14 Patients and Brief Review of Literature. Clujul Med..

[117] Mihailovici A., Rottenstreich M., Kovel S., Wassermann I., Smorgick N., Vaknin Z. (2017). Endometriosis-Associated Malignant Transformation in Abdominal Surgical Scar: A PRISMA-Compliant Systematic Review. Medicine.

[118] Maxwell G.L., Risinger J.I., Gumbs C., Shaw H., Bentley R.C., Barrett J.C., Berchuck A., Futreal P.A. (1998). Mutation of the PTEN Tumor Suppressor Gene in Endometrial Hyperplasias. Cancer Res..

[119] Tashiro H., Blazes M.S., Wu R., Cho K.R., Bose S., Wang S.I., Li J., Parsons R., Ellenson L.H. (1997). Mutations in PTEN Are Frequent in Endometrial Carcinoma but Rare in Other Common Gynecological Malignancies. Cancer Res..

[120] Tamura M., Gu J., Matsumoto K., Aota S., Parsons R., Yamada K.M. (1998). Inhibition of Cell Migration, Spreading, and Focal Adhesions by Tumor Suppressor PTEN. Science.

[121] Tamura M., Gu J., Takino T., Yamada K.M. (1999). Tumor Suppressor PTEN Inhibition of Cell Invasion, Migration, and Growth: Differential Involvement of Focal Adhesion Kinase and p130Cas. Cancer Res..

[122] Sulzmaier F.J., Jean C., Schlaepfer D.D. (2014). FAK in Cancer: Mechanistic Findings and Clinical Applications. Nat. Rev. Cancer.

[123] Mikuła-Pietrasik J., Uruski P., Tykarski A., Książek K. (2018). The Peritoneal “Soil” for a Cancerous “Seed”: A Comprehensive Review of the Pathogenesis of Intraperitoneal Cancer Metastases. Cell. Mol. Life Sci..

[124] Williams C.B., Yeh E.S., Soloff A.C. (2016). Tumor-Associated Macrophages: Unwitting Accomplices in Breast Cancer Malignancy. NPJ Breast Cancer.

[125] Morris P.G., Hudis C.A., Giri D., Morrow M., Falcone D.J., Zhou X.K., Du B., Brogi E., Crawford C.B., Kopelovich L. (2011). Inflammation and Increased Aromatase Expression Occur in the Breast Tissue of Obese Women with Breast Cancer. Cancer Prev. Res..

[126] Amant F., Moerman P., Neven P., Timmerman D., Van Limbergen E., Vergote I. (2005). Endometrial Cancer. Lancet.

[127] Kitson S., Ryan N., MacKintosh M.L., Edmondson R., Duffy J.M., Crosbie E.J. (2018). Interventions for Weight Reduction in Obesity to Improve Survival in Women with Endometrial Cancer. Cochrane Database Syst. Rev..

[128] Potischman N., Hoover R.N., Brinton L.A., Siiteri P., Dorgan J.F., Swanson C.A., Berman M.L., Mortel R., Twiggs L.B., Barrett R.J. (1996). Case—Control Study of Endogenous Steroid Hormones and Endometrial Cancer. J. Natl. Cancer Inst..

[129] Bloom H.J., Richardson W.W. (1957). Histological Grading and Prognosis in Breast Cancer; a Study of 1409 Cases of Which 359 Have Been Followed for 15 Years. Br. J. Cancer.

[130] Dunnwald L.K., Rossing M.A., Li C.I. (2007). Hormone Receptor Status, Tumor Characteristics, and Prognosis: A Prospective Cohort of Breast Cancer Patients. Breast Cancer Res..

[131] Nakshatri H., Bhat-Nakshatri P., Martin D.A., Goulet R.J., Sledge G.W. (1997). Constitutive Activation of NF-kappaB during Progression of Breast Cancer to Hormone-Independent Growth. Mol. Cell. Biol..

[132] Dumont J.A., Bitonti A.J., Wallace C.D., Baumann R.J., Cashman E.A., Cross-Doersen D.E. (1996). Progression of MCF-7 Breast Cancer Cells to Antiestrogen-Resistant Phenotype Is Accompanied by Elevated Levels of AP-1 DNA-Binding Activity. Cell Growth Differ..

[133] Brooks P.C., Strömblad S., Sanders L.C., von Schalscha T.L., Aimes R.T., Stetler-Stevenson W.G., Quigley J.P., Cheresh D.A. (1996). Localization of Matrix Metalloproteinase MMP-2 to the Surface of Invasive Cells by Interaction with Integrin Alpha v Beta 3. Cell.

[134] Stein B., Baldwin A.S., Ballard D.W., Greene W.C., Angel P., Herrlich P. (1993). Cross-Coupling of the NF-Kappa B p65 and Fos/Jun Transcription Factors Produces Potentiated Biological Function. EMBO J..

[135] Pino M., Galleguillos C., Torres M., Sovino H., Fuentes A., Boric M.A., Johnson M.C. (2009). Association between MMP1 and MMP9 Activities and ICAM1 Cleavage Induced by Tumor Necrosis Factor in Stromal Cell Cultures from Eutopic Endometria of Women with Endometriosis. Reproduction.

[136] Manthe R.L., Muro S. (2017). ICAM-1-Targeted Nanocarriers Attenuate Endothelial Release of Soluble ICAM-1, an Inflammatory Regulator. Bioeng. Transl. Med..

[137] Deem T.L., Cook-Mills J.M. (2004). Vascular Cell Adhesion Molecule 1 VCAM-1Activation of Endothelial Cell Matrix Metalloproteinases: Role of Reactive Oxygen Species. Blood.

[138] Lu X., Lu D., Scully M., Kakkar V. (2008). ADAM Proteins - Therapeutic Potential in Cancer. Curr. Cancer Drug Targets.

[139] Pires B.R.B., Silva R.C.M.C., Ferreira G.M., Abdelhay E. (2018). NF-kappaB: Two Sides of the Same Coin. Genes.

[140] Tsai H.-W., Huang M.-T., Wang P.-H., Huang B.-S., Chen Y.-J., Hsieh S.-L. (2017). Decoy Receptor 3 Promotes Cell Adhesion and Enhances Endometriosis Development. J. Pathol..

[141] Pitti R.M., Marsters S.A., Lawrence D.A., Roy M., Kischkel F.C., Dowd P., Huang A., Donahue C.J., Sherwood S.W., Baldwin D.T. (1998). Genomic Amplification of a Decoy Receptor for Fas Ligand in Lung and Colon Cancer. Nature.

[142] Ge Z., Sanders A.J., Ye L., Wang Y., Jiang W.G. (2011). Expression of Death Decoy Receptor-3 DcR3in Human Breast Cancer and Its Functional Effects on Breast Cancer Cells in Vitro. J. Exp. Ther. Oncol..

[143] Wagner B.J., Löb S., Lindau D., Hörzer H., Gückel B., Klein G., Glatzle J., Rammensee H.-G., Brücher B.L., Königsrainer A. (2011). Simvastatin Reduces Tumor Cell Adhesion to Human Peritoneal Mesothelial Cells by Decreased Expression of VCAM-1 and β1 Integrin. Int. J. Oncol..

[144] Banks R.E., Gearing A.J., Hemingway I.K., Norfolk D.R., Perren T.J., Selby P.J. (1993). Circulating Intercellular Adhesion Molecule-1 ICAM-1), E-Selectin and Vascular Cell Adhesion Molecule-1 VCAM-1in Human Malignancies. Br. J. Cancer.

[145] Heinzelmann-Schwarz V.A., Gardiner-Garden M., Henshall S.M., Scurry J., Scolyer R.A., Davies M.J., Heinzelmann M., Kalish L.H., Bali A., Kench J.G. (2004). Overexpression of the Cell Adhesion Molecules DDR1, Claudin 3, and Ep-CAM in Metaplastic Ovarian Epithelium and Ovarian Cancer. Clin. Cancer Res..

[146] Gogali A., Charalabopoulos K., Zampira I., Konstantinidis A.K., Tachmazoglou F., Daskalopoulos G., Constantopoulos S.H., Dalavanga Y. (2010). Soluble Adhesion Molecules E-Cadherin, Intercellular Adhesion Molecule-1, and E-Selectin as Lung Cancer Biomarkers. Chest.

[147] Christou N., Perraud A., Blondy S., Jauberteau M., Battu S., Mathonnet M. (2017). E-cadherin: A Potential Biomarker of Colorectal Cancer Prognosis Review). Oncol. Lett..

[148] Magrina J.F. (2002). Complications of Laparoscopic Surgery. Clin. Obstet. Gynecol..

[149] Johnston J.L., Reid H., Hunter D. (2015). Diagnosing Endometriosis in Primary Care: Clinical Update. Br. J. Gen. Pract..

[150] Fassbender A., Vodolazkaia A., Saunders P., Lebovic D., Waelkens E., De Moor B., D’Hooghe T. (2013). Biomarkers of Endometriosis. Fertil. Steril..

